# Fabrication and Characterization of Polysaccharide Ion Gels with Ionic Liquids and Their Further Conversion into Value-Added Sustainable Materials

**DOI:** 10.3390/biom5010244

**Published:** 2015-03-18

**Authors:** Akihiko Takada, Jun-ichi Kadokawa

**Affiliations:** 1 Institute for Materials Chemistry and Engineering, Kyushu University, Kasuga Koen 6-1, Kasuga, Fukuoka 816-8581, Japan; E-Mail: takada@mm.kyushu-u.ac.jp; 2 Graduate School of Science and Engineering, Kagoshima University, 1-21-40 Korimoto, Kagoshima 890-0065, Japan; 3 Research Center for Environmentally Friendly Materials Engineering, Muroran Institute of Technology, 27-1 Mizumoto-cho, Muroran, Hokkaido 050-8585, Japan

**Keywords:** polysaccharides, ion gels, ionic liquids, sustainable materials

## Abstract

A review of the fabrication of polysaccharide ion gels with ionic liquids is presented. From various polysaccharides, the corresponding ion gels were fabricated through the dissolution with ionic liquids. As ionic liquids, in the most cases, 1-butyl-3-methylimidazolium chloride has been used, whereas 1-allyl-3methylimidazolium acetate was specifically used for chitin. The resulting ion gels have been characterized by suitable analytical measurements. Characterization of a pregel state by viscoelastic measurement provided the molecular weight information. Furthermore, the polysaccharide ion gels have been converted into value-added sustainable materials by appropriate procedures, such as exchange with other disperse media and regeneration.

## 1. Introduction

**Figure 1:**
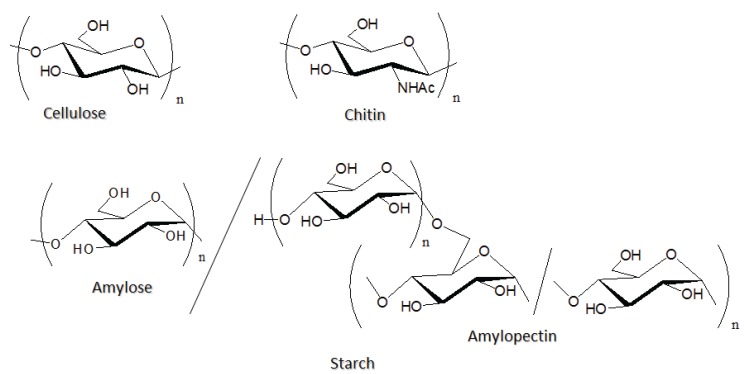
Structures of cellulose, chitin, and starch.

**Figure 2:**
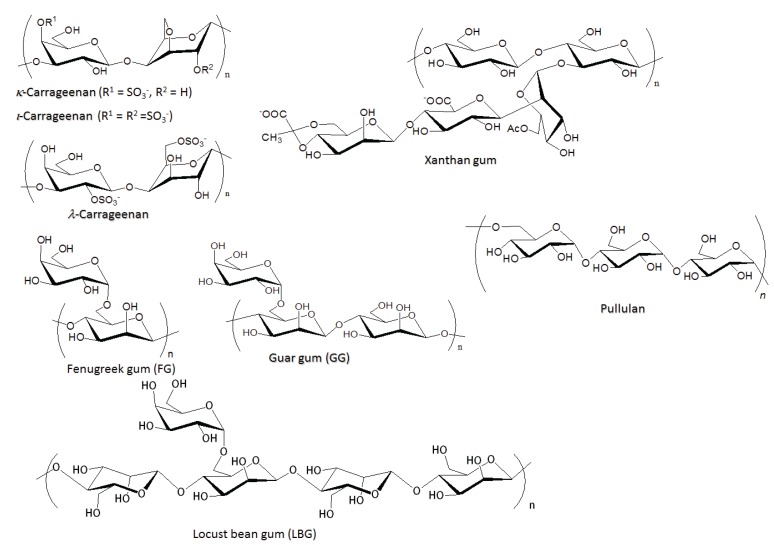
Structures of hydrocolloid polysaccharides, which are used in the fabrication of ion gels.

Polysaccharides are widely distributed in nature and act as important *in vivo* substrates, such as structural material and energy provider [1]. They are also regarded as representative biomass resources and are used as the components in functional bio-based materials because of their eco-friendly and biodegradable properties [2,3]. Cellulose, starch, and chitin are the most abundant natural polysaccharides, which are found in plants, insects, microorganisms, and so on [4,5,6,7,8]. Cellulose and chitin act as structural materials present in the cell walls of plants and the exoskeletons of crustaceans, shellfishes, and insects, respectively. Accordingly, cellulose and chitin have similar structures, in which the former is composed of β-(1→4)-linked d-glucose residues and the latter is an aminopolysaccharide consisting of *N*-acetyl-d-glucosamine units linked through β-(1→4)-glycosidic linkages (Figure 1). Starch consists of amylose and amylopectin and both of which are glucose polymers [8]. Amylose is s linear polysaccharide composed of α-(1→4)-linked d-glucose residues and amylopectin has a branched structure of such α-(1→4)-linked glucose chains interlinked through α-(1→6)-glycosidic bonds (Figure 1). Because of the opposite stereofashion of starch from cellulose, the role of the former is completely different from the latter, which is an energy resource. Besides such abundant polysaccharides, there are a number of polysaccharides in nature, which have specific structures composed of one or multiple kinds of monosaccharide residues [1]. Furthermore, such polysaccharides sometimes consist of complicated branched or grafted structures with the different kinds of saccharide chains. Because the polysaccharides form highly viscous aqueous solutions, they have been used as hydrocolloids for a stabilizer, a viscous agent, and a structure provider in food industries (Figure 2) [9]. Polysaccharides often show a lack of solubility and processability because of numerous intra- and intermolecular hydrogen bonds among hydroxy groups in saccharide residues.

Over the past decade, ionic liquids (ILs) have been noted as powerful solvents for polysaccharides [10,11,12,13,14] since Rogers *et al.* reported the dissolution of cellulose with IL, 1-butyl-3-methylimidazolium chloride (BMIMCl), in relatively high concentrations [15]. ILs are low-melting point molten salts, generally defined as exhibiting liquid states at temperatures below the boiling point of water [16,17,18]. The property is owing to that the liquid state is thermodynamically favorable because of the large size and conformational flexibility of the ions, in which these behaviors lead to small lattice enthalpies and large entropy changes that favor the liquid state. In 1934, it had already been discovered that a molten *N*-ethylpyridinium chloride, in the presence of nitrogen-containing bases, dissolved cellulose [19]. This was probably the first example of the cellulose dissolution with IL-type solvents. However, this was considered to be of little practical value at the time because of the concept of ILs had not been put forward. Then, the aforementioned dissolution study of cellulose with BMIMCl by Rogers in 2002 opened up a new way for the development of a class of cellulose solvent systems. Since this study, ILs have begun being recognized as good solvents for cellulose and other polysaccharides, and thus used as media for material processing, such as derivatization, modification, and regeneration [12,20,21,22,23].

**Figure 3:**
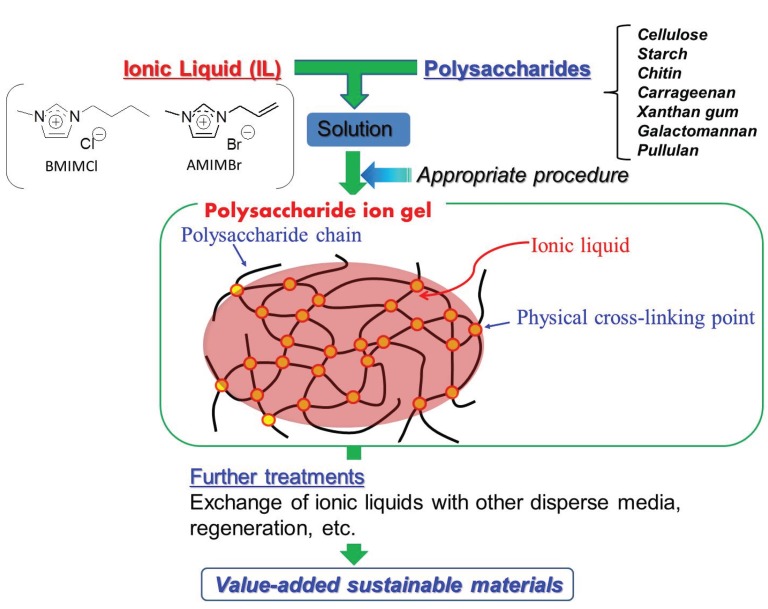
Schematic diagram for procedures of fabrication of polysaccharide ion gels with IL and conversion into value-added sustainable materials.

In recent years, we have extensively used ILs as disperse media in polysaccharide-based gels, so-called “ion gels”, which include ILs in the polysaccharide network matrixes (Figure 3) [24,25,26], besides the traditional use as solvents mentioned above, it was found that cellulose formed an ion gel with BMIMCl [27]. Furthermore, the conversion of the ion gels of various polysaccharides into value-added sustainable materials has also been performed by suitable treatments, such as exchange of disperse media and regeneration (Figure 3) [28,29,30]. In this article, we presented a review of these studies with sections separated in accordance with the kinds of polysaccharides.

## 2. Cellulose Ion Gels

**Figure 4:**
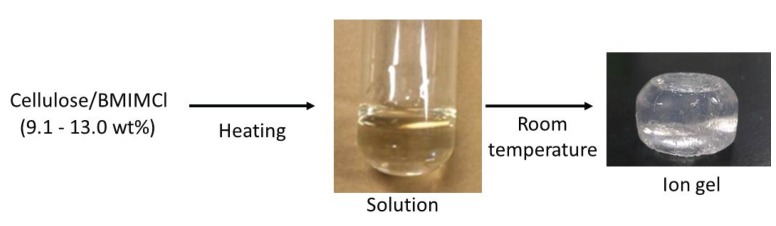
Procedure for fabrication of cellulose ion gel with 1-butyl-3-methylimidazolium chloride (BMIMCl).

**Figure 5:**
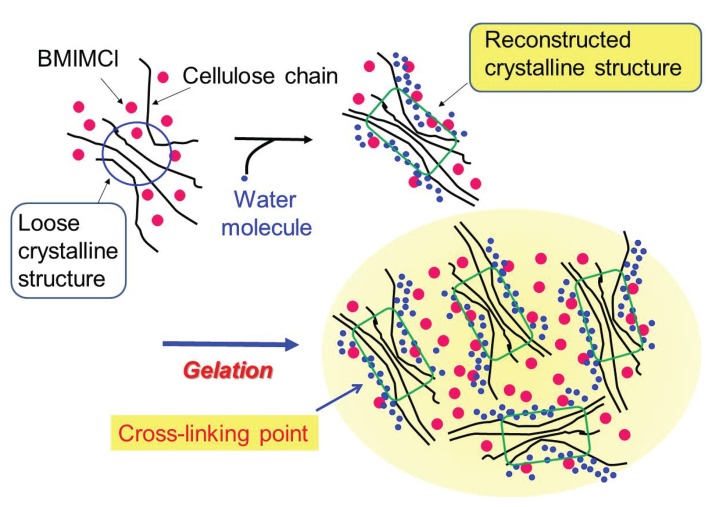
Plausible mechanism for formation of cellulose ion gel.

When solutions of cellulose in BMIMCl (9.1–13.0 wt%), which were prepared by heating the mixtures, were left standing at room temperature for seven days, the gelation was progressed with exclusion of excess BMIMCl from the gel matrix (Figure 4) [27]. The resulting gel was purified by washing with ethanol and dried under reduced pressure. Thus, a flexible and manipulatable gel was obtained, which was characterized by powder X-ray diffraction (XRD), thermogravimetric analysis (TGA), and elemental analysis measurements. The XRD profile of the ion gel indicated that the crystalline structure of cellulose was mostly disrupted, but still slightly remained. The TGA profile of the ion gel showed two step weight losses at temperatures below 100 °C and higher than 250 °C. The former and latter are reasonably attributed to evaporation of water and degradation of cellulose, respectively. This data strongly indicated that the ion gel was composed not only of cellulose and BMIMCl, but also of water (*ca.* 15 wt%). The elemental analysis result also supported the presence of the large amount of water in the gel. The analytical results suggested the fact that the absorption of water into the solution, due to the strong hygroscopic nature of BMIMCl, probably caused the gelation of cellulose with BMIMCl. Because BMIMCl does not dissolve cellulose perfectly from a molecular point of view, aggregates of crystalline parts partially present among cellulose molecules were formed by gradually absorbing water into the solution, owing to its insolubility in water. Indeed, the colorless solution tuned into turbid during the gelation because of the formation of the aggregates. The aggregates probably acted as cross-linking points for the formation of the ion gel (Figure 5).

**Figure 6:**
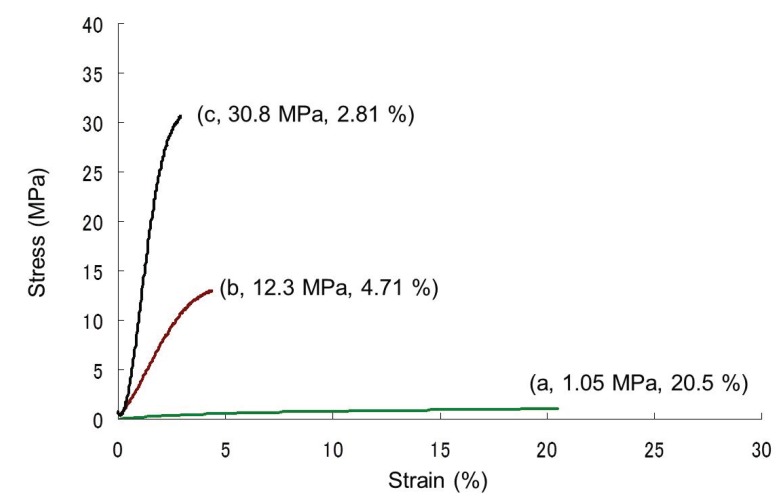
Stress-strain curves of regenerated cellulose materials obtained from cellulose ion gels by Soxhlet extraction with ethanol for 0.5 h (**a**); 1 h (**b**); and 4 h (**c**) (tensile mode).

Soxhlet extraction of the ion gels with ethanol gave the regenerated cellulose materials with different BMIMCl contents. With prolonged extraction times, the contents of BMIMCl in the products decreased as follows; cellulose: BMIMCl (mol/mol) = 1:1.34 by 0.5 h extraction and 1:0.23 by 1 h extraction, and by 4 h extraction, regenerated cellulose with little BMIMCl was obtained. The tensile testing indicated that the materials with higher BMIMCl contents showed elastic nature, whereas the hard nature was exhibited from the material with less BMIMCl content (Figure 6). These results strongly suggested the influence of the BMIMCl content for the mechanical property of the regenerated celluloses.

## 3. Chitin Ion Gels

**Figure 7:**
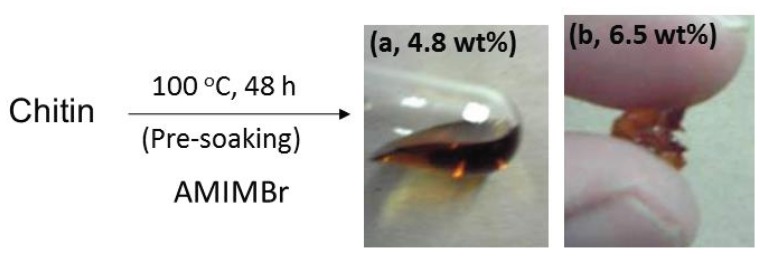
Dissolution (**a**) and gelation (**b**) of chitin with 1-allyl-3-methylimidazolium bromide (AMIMBr).

Different from cellulose, only a few examples have been reported on the dissolution of chitin with ILs [31,32,33,34]. For example, it has been reported that 1-allyl-, 1-butyl-, and 1-ethyl-3-methylimidazolium acetates dissolved chitin in certain concentrations [31,35,36]. We also studied on the dissolution of chitin with ILs. For the dissolution study, we have noted the previous publication on synthesis of polyamides and polyimides using ILs with the imidazolium bromide structure as reaction media [37]. This investigation inspired us to employ the same kind of ILs for the dissolution of chitin because chitin forms strong hydrogen bonds by the –N-C=O groups of acetamido causing the solubility problem, same as the case of polyamides and polyimides. In imidazolium-type ILs with a bromide counter anion tested, consequently, we found only 1-allyl-3-methylimidazolium bromide (AMIMBr) dissolved chitin in concentrations up to 4.8 wt% by heating the mixture at 100 °C for 48 h (Figure 7) [38,39]. The SEM observation of the resulting liquid clearly indicated the complete dissolution of chitin with AMIMBr. The IR, TGA, and XRD results of the regenerated chitin from the solution, as well as the ^1^H NMR and gel permeation chromatographic data of its hexanoyl derivative suggested that the degradation and depolymerization of chitin did not frequently occur during the dissolution process. Although the reason why AMIMBr specifically dissolved chitin is not yet clear, a combination of an allyl substituent and a bromide counter anion on the imidazolum probably contributes to exhibit its dissolution ability. Moreover, when a mixture of a higher amount of chitin with AMIMBr (6.5 wt%) was left standing at room temperature, followed by heating at 100 °C, it turned into an ion gel. As shown Figure 7, indeed, the 6.5 wt% chitin with AMIMBr showed the viscous and manipulatable natures, whereas the 4.8 wt% chitin with AMIMBr flowed upon leaning a test tube. The dynamic rheological measurement, however, showed that both the 4.8 wt% and 6.5 wt% liquids of chitin with AMIMBr behaved as the weak gels [39]. In the mixture of a higher amount of chitin with AMIMBr, the chitin chains probably interact with each other to spontaneously construct a network structure with junction zones, which contributes to the formation of the ion gel.

**Figure 8:**
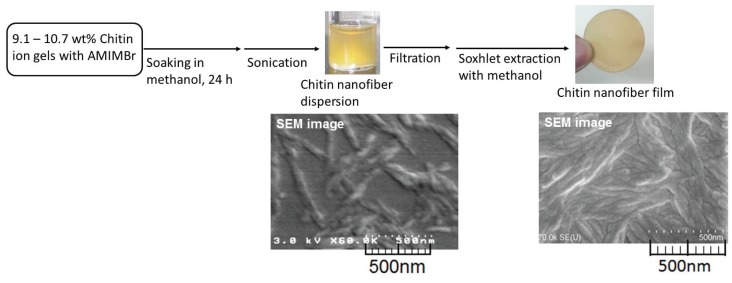
Procedures for fabrication of self-assembled chitin nanofiber dispersion and film, and their SEM images.

When the chitin ion gels with AMIMBr (9.1–10.7 wt%) were soaked in methanol at room temperature for 24 h to slowly regenerate chitin, followed by sonication, dispersions were produced (Figure 8) [40,41]. The SEM image of a spin-coated sample obtained by dilution of the dispersion showed the nanofiber morphology with *ca.* 20–60 nm in width and several hundred nm in length, indicating the self-assembled formation of chitin nanofibers by the regenerative bottom-up approach from the ion gel. The isolation of the chitin nanofibers from the dispersion by filtration gave a film (Figure 8). The SEM image of the film exhibited a surface morphology of highly entangled nanofibers. Such entangled structure from the nanofibers probably contributed to the formability of the film.

**Figure 9:**
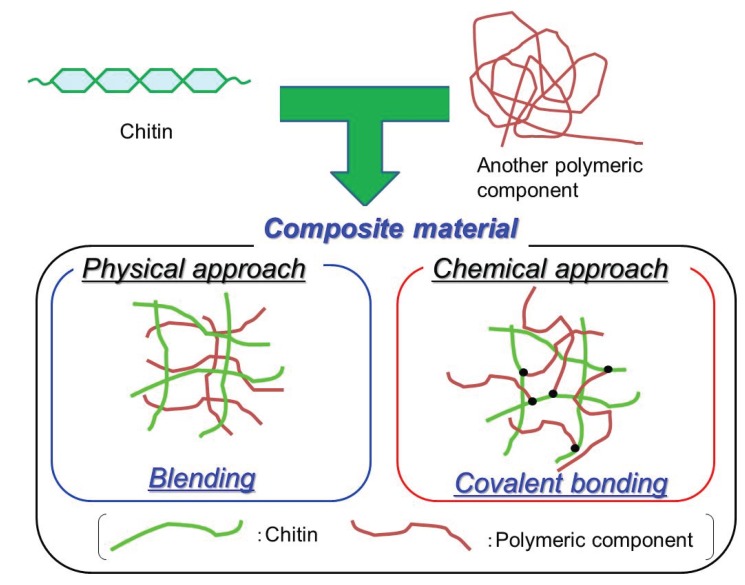
Physical and chemical approaches for fabrication of composite materials of chitin with another polymeric component.

As one of the possible applications of self-assembled chitin nanofibers, attempts have been made to fabricate composite materials with other polymeric components. Two kinds of processes, that is, physical and chemical approaches, have been conducted to fabricate composite materials from the nanofibers. In the former case, nanofibers and polymeric components are physically interacted to construct composites, whereas the latter approach exploits covalent linkages between nanofibers and polymers, leading to compatibility (Figure 9). In general, the latter approach requires the more complicated procedure, including appropriate chemical reactions, than the former approach. By the physical approach, poly(vinyl alcohol) and carboxymethyl cellulose have been compatibilized with self-assembled chitin nanofibers by means of co-regeneration and electrostatic interaction, respectively, to obtain composite films [40,42]. By the chemical approach, surface-initiated ring-opening (co)polymerizations of cyclic monomers from self-assembled chitin nanofiber films with the appropriate initiating groups have been conducted to fabricate composite films covalently grafting polyester and polypeptide chains on the nanofibers [43,44]. Surface-initiated atom-transfer radical polymerization (ATRP) of an acrylate monomer from the self-assembled chitin nanofiber film, which carried initiating groups for ATRP, was also conducted to give composite films [45].

## 4. Hydrocolloid Polysaccharide Ion Gels

As aforementioned, many kinds of polysaccharides from various sources, such as plants, animals, seaweeds, and bacteria, have been known as food hydrocolloids besides the abundant polysaccharides like cellulose and chitin [9]. However, there have not been many reports on the use of hydrocolloid polysaccharides as the components of practical materials. To provide practical materials from such polysaccharides, therefore, the fabrication of ion gels of several hydrocolloid polysaccharides has also been attempted.

**Figure 10:**
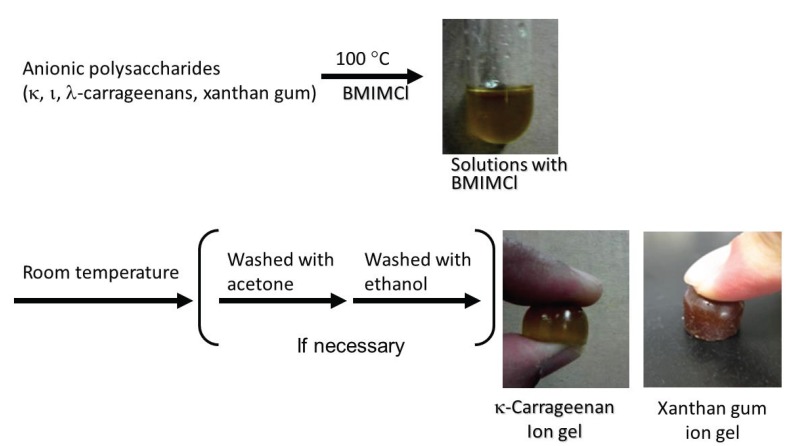
Procedure for fabrication of anionic polysaccharide ion gels with BMIMCl.

Carrageenan is a water-soluble phycocolloid extracted from red algae and is used mostly as a stabilizer and a structural provider in food and ice cream industries [46]. Three major varieties of carrageenans, *i.e.*, κ-, λ-, and ι-carrageenans, are known. They are anionic polysaccharides because of the presence of sulfate groups and differ in their number and position on the repeating units (Figure 2). We reported the gelling system of these carrageenans with BMIMCl (Figure 10) [47]. For the fabrication of ion gels, first, carrageenans were dissolved with BMIMCl (13 wt%) at 100 °C for 10 h with stirring. When the solutions were cooled to room temperature, ion gels were obtained, which could suitably be manipulated after standing for 12 h upon addition of acetone and excess IL excluding from the gels were washed out with ethanol. κ-Carrageenan formed a hard gel, whereas the other carrageenans formed soft gels. It should be noted that the κ-carrageenan gel is tough in nature and hence not able to sustain more strain, unlike the other two carrageenan gels, which had soft natures [9].

**Figure 11:**
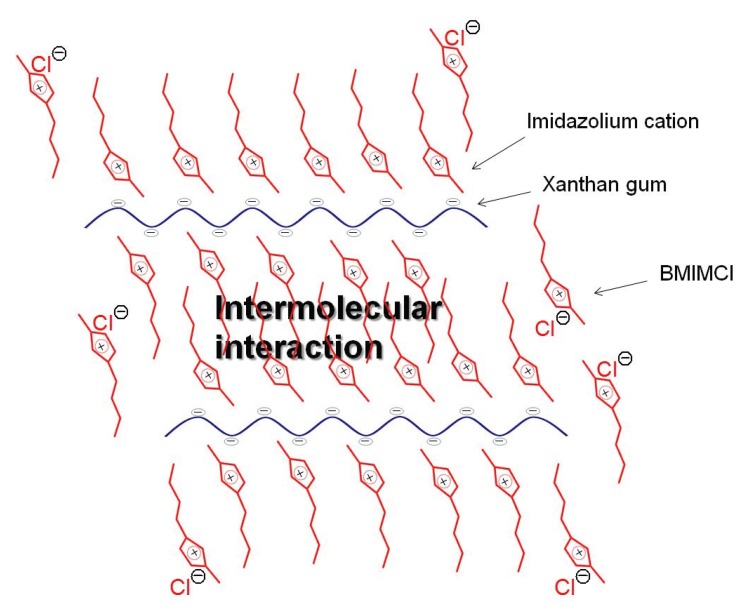
Plausible associate structure of xanthan gum and BMIMCl in ion gel.

Xanthan gum, which is also a representative hydrocolloid polysaccharide produced by *Xanthomonas canpestris*, is composed of cellulose-type main chain (β-(1→4)-glucan) with trisaccharide side chains attached to alternating glucose units in the main chain (Figure 2) [48]. The side chains are partially acetylated and pyruvated. Because the side chains include carboxylate groups, xanthan gum is an anionic polysaccharide. It was found that solutions of xanthan gum (9.1–50 wt%), which were prepared by heating the mixtures at 100 °C, totally turned into the gel form by standing at room temperature for 20 min (Figure 10) [49,50]. Similar to the fabrication of the chitin ion gel with AMIMBr as mentioned above, the present procedure for the gelation was also very simple, compared with that for the ion gels from cellulose and carrageenans with BMIMCl, where long duration or treatment with appropriate organic media was required to generate the stable ion gels. All the xanthan gum ion gels with the different contents exhibited good mechanical properties under compressive mode. The 9.1 wt% ion gel exhibited very elastic nature and the properties gradually changed to be tougher with increasing of the content of xanthan gum in the gels. The ion gels had sufficient strength to apply to tensile testing, too. The 9.1 and 16.7 wt% gels showed good elasticity, which were elongated to strain values of *ca.* 400%. In contrast, the 50 wt% gel showed very hard nature with the elastic modulus value of 14.8 MPa and the tensile strength value of 2.1 MPa. Furthermore, the xanthan gum ion gels exhibited thermally induced shape-memory behavior. First, a designed permanent shape of the gel was fabricated by heating-cooling process using an appropriate mold. Then, the gel was softened and deformed by heating at around 50 °C, and then intended temporary shape was fixed by cooling at room temperature. When the resulting gel was left standing at around 50 °C, its temporary shape gradually returned back and almost recovered to its permanent shape. It was supposed that the shape memory behavior of the ion gel was generated by the effect of ion-exchange of the carboxylate metal salts in xanthan gum with BMIMCl during the gel formation and also by the rigid nature of the xanthan gum main chain. Indeed, the analytical data of the ion gel by the NMR, XRD, and UV-vis measurements suggested the formation of specific association of BMIMCl in the ion gel, which was constructed on the basis of the regularly ordered intermolecular interaction of imidazolium counter cations of xanthan gum with other BMIMCl present in the gel because of the rigidity of the main chain (Figure 11). The specific association probably contributed to appearance of the shape-memory behavior of the ion gel.

The 9.1 wt% ion gel was converted into a hydrogel of xanthan gum by soaking it in water (1.15 g/100 mL). The water content of the obtained hydrogel was 94.1 wt%. The hydrogel was then soaked in 0.2 mol/L CaCl_2_ aqueous solution to produce an ionically cross-linked hydrogel with divalent Ca^2+^ cations. The water content of the product was 85.2 wt%, indicating shrinkage by ionic cross-linking. In addition, this material showed reversible swelling-shrinking behavior. When the ionically cross-linked hydrogel was soaked in water for one day, it was gradually swollen. Soaking the swollen hydrogel in 0.2 mol/L CaCl_2_ aqueous solution for one day caused rapid shrinkage. The swelling-shrinkage cycles of the hydrogel were repeated two more times. These results indicated that the present xanthan gum hydrogel exhibited the salt concentration-induced responsive property.

**Figure 12:**
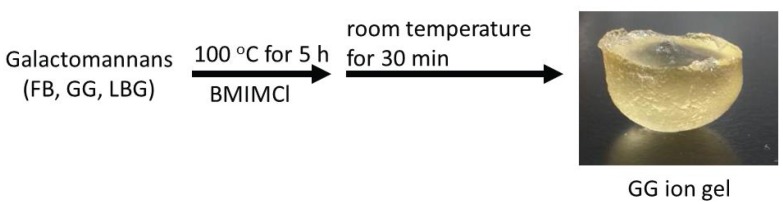
Procedure for fabrication of galactomannan ion gels with BMIMCl.

The gel formation of hydrocolloid polysaccharides with BMIMCl was not limited to the anionic polysaccharides, but was extended to neutral polysaccharides, such as galactomannans. They are also representative food hydrocolloids, consisting of a β-(1→4)-linked mannopyranose main chain with a branched α-galactopyranose unit at the 6-position [51]. The proportions of galactose and mannose residues are depending on the source. Three major galactomannans, that is, fenugreek gum (FG), guar gum (GG), and Locust bean gum (LBG), have an average galactose/mannose ratios of *ca.* 1:1, 1:1.8, and 1:3.5, respectively (Figure 2). When solutions of GG with BMIMCl (9.1–28.6 wt%), which were prepared by heating the mixtures for 100 °C for 5 h, were left standing at room temperature for 30 min, they totally turned into the gelling form (Figure 12) [52,53]. The mechanical properties of the ion gels under both compressive and tensile modes depended on the contents of GG in the ion gels. From the other galactomannans, FG and LBG, the corresponding ion gels were also produced by the same procedures [54].

The GG ion gels were converted into films by compression technique (60 °C, 4 MPa), followed by immersing in ethanol. The mechanical properties of the resulting films depended on the contents of GG in the films. The film showed the temperature-induced shaping ability, in which it became hard upon heating and returned back to soft nature upon cooling. The TGA and XRD analyses of the film indicated that this property was owing to construction and disruption of crystalline structures of GG in the film by evaporation and absorption of water during the heating-cooling process. Moreover, the ion gels were converted into porous materials by soaking it in ethanol and water, followed by lyophilization. Porous materials are known as high performance column phases for liquid chromatography.

The unique fluorescent material was obtained by exploiting the gelling system of GG with BMIMCl [55]. A mixture of GG with Rhodamine 6G, a representative red fluorescent dye, in BMIMCl was heated at 100 °C, followed by cooling to room temperature, giving rise to the GG ion gel containing Rhodamine 6G. The fluorescence spectra of the ion gel showed the emissions of Rhodamine 6G at *ca.* 608 nm by excitation at 260–620 nm. On the other hand, no emissions of BMIMCl, which had typically been observed at around 450–600 nm, were detected. These results indicated the occurrence of fluorescence resonance-energy-transfer from the BMIMCl donor to the Rhodamine 6G acceptor in the ion gel. Indeed, it was further revealed that the emission peaks in the fluorescence spectra of the GG ion gel with BMIMCl excited at various wavelengths certainly overlapped with the absorption peak of Rhodamine 6G at 545 nm in its solution in BMIMCl.

Using IL solutions of polysaccharides as a pregel state of IL gels, we can evaluate the solution properties. IL solvents dissolving polysaccharides generally have a high viscosity, which gives us the advantage that we can measure fast molecular relaxations by viscoelastic measurement because molecular motions become slower in solvents with higher viscosity. In many cases, even a clear IL solution of a polysaccharide, such as cellulose, is obtained at a glance, while it does not dissolve perfectly from a molecular point of view as mentioned above. By viscoelastic measurement on the solution, thus, we can evaluate whether we prepare a solution with molecular dispersion state [56], which in turn we potentially estimate a molecular weight (MW) of the polysaccharide by viscoelastic measurement.

**Figure 13:**
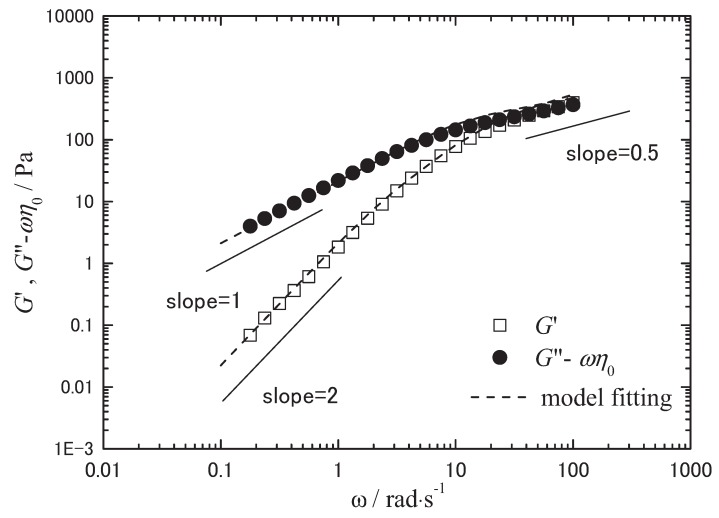
Viscoelastic measurement results on BMIMCl solution of a standard pullulan (MW = 4.04 × 10^5^, MWD =1.13). *G*', *G*", and ω are storage modulus, loss modulus and angular frequency, respectively. *C* = 4.0 × 10^−2^ (g/cm^3^), *T* = 25 °C. The broken lines show fitting curves by the revised Rouse model.

For this investigation, we selected pulluan, also a well-known hydrocolloid, as the suitable polysaccharide, because standard pullulan samples with controlled MWs and narrow molecular weight distribution (MWD) are commercially available [57]. Pullulan is an extracellular polysaccharide produced by the fungus *Aureobasidium pullulans* (*Pullularia pullulans*). It is composed of a linear chain of glucose units that alternates regularly between one α-(1→6)- and two α-(1→4)-glycoside linkages (Figure 2) [58]. Figure 13 shows a result of the viscoelastic measurements on a BMIMCl solution of pullulan with a narrow MWD (MW = 4.04 × 10^5^, MWD = 1.13) as a standard sample of polysaccharide [59]. In this figure, the experimental results were compared with the theoretical curves by a revised Rouse model, in which a slow relaxation mode was added into the original model [60] by Osaki *et al.* [61,62].

$$G'(\omega )\;=\;{\left(\frac{CRT}{M}\sum_{p=1}^{N}\frac{\omega \tau_{P}}{1+\omega^{2}{\tau_{P}}^{2}}\right)}_{Rouse}+{\left(G_{LT}\frac{\tau_{LT}\omega }{1+{\tau_{LT}}^{2}\omega^{2}}\right)}_{LongTerm}$$

$$G'(\omega )\;={\left(\frac{CRT}{M}\sum_{p=1}^{N}\frac{\omega^{2}{\tau_{P}}^{2}}{1+\omega^{2}{\tau_{P}}^{2}}\right)}_{Rouse}+{\left(G_{LT}\frac{{\tau_{LT}}^{2}\omega^{2}}{1+{\tau_{LT}}^{2}\omega^{2}}\right)}_{LongTerm}+\omega \eta_{0}$$

$$\tau_{p}=\frac{6\eta^{o}M}{\pi^{2}p^{2}CRT},\tau_{LT}=P\tau_{1}\;,G_{LT}=QC[\eta ]\frac{CRT}{M}$$

where *C* is concentration, *R* the gas constant, *T* temperature, *M* molecular weight, η_0_ solvent viscosity, and [η] intrinsic viscosity. *P* and *Q* are non-dimensional fitting parameters, which have almost constant values in the Rouse *C* region of behavior for a same system.

The coincidence of theoretical curves and the experimental results implies that we can estimate MW by viscoelastic measurements with appropriate conditions. This estimation should be available in the C range where viscoelastic behavior of the solutions resembles the Rouse model with hydrodynamic effects shielded. Roughly speaking, the C range is higher than the overlapping concentration (around *C*[η] = 2.5) [61,62] and lower than the C where entanglement effect starts to appear. In even lower C region, if we introduce a relaxation distribution parameter α, we can fit the data by Rouse-Zimm model [61,62].

The same type of fitting was available for IL solutions of cellulose and other biopolymers, too [63]. Using such techniques, studies on IL solutions should provide us molecular information of polysaccharides. And it is expected that we can elucidate more detailed molecular interpretation on gelation behaviors, such as gelation dynamics or gelation properties.

## 5. Binary Polysaccharide Ion Gels Containing Cellulose

**Figure 14:**
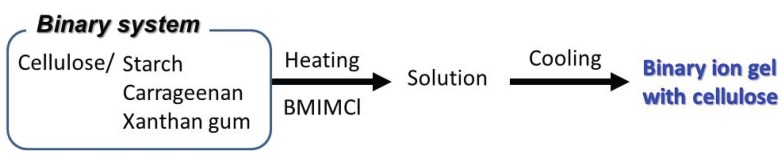
Procedure for fabrication of binary ion gels with BMIMCl.

The preparation procedure for the cellulose ion gel with BMIMCl was extended to fabricate some binary gelling systems with the other polysaccharides (Figure 14). For example, when a solution of cellulose and potato starch (9.1 and 4.8 wt%, respectively) in BMIMCl, which was prepared by heating a mixture at 100 °C for 24 h, was left standing at room temperature for several days, a binary ion gel was fabricated with excluding excess BMIMCl [64]. The XRD profile of the resulting gel did not show the obvious diffraction peaks assignable to crystalline structures of cellulose and starch, suggesting that crystalline structures of the polysaccharides were mostly disrupted in the gel. The analytical data of the gel indicated to conceive the similar gelation process as that for the aforementioned cellulose ion gel with BMIMCl.

The homogeneous mixture of cellulose and starch (9.1 wt% each) in BMIMCl was very viscous, and thus could be handled up from the surface. Then, the resulting fine linear material was soaked in acetone for regeneration to fabricate a binary polysaccharide fiber. The SEM image of the regenerated binary material exhibited the fiber morphology with *ca.* 100–200 μm in width. The XRD result of the fiber indicated that crystalline structures of cellulose and starch were not reconstructed, suggesting good compatibility of the two polysaccharides in the fibrous material.

**Figure 15:**
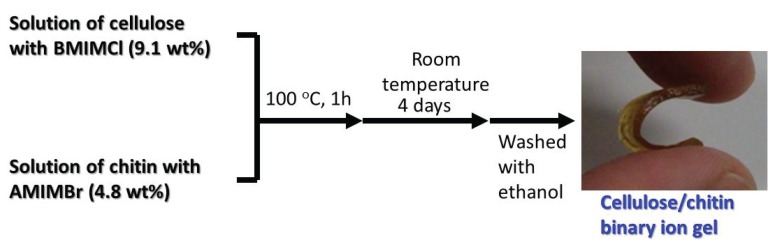
Procedure for fabrication of cellulose/chitin binary ion gel with ILs, BMIMCl and AMIMBr.

The fabrication of a cellulose/chitin binary ion gel using the two ILs, BMIMCl and AMIMBr, was also performed (Figure 15) [65]. First, a 9.1 wt% cellulose solution with BMIMCl and a 4.8 wt% chitin solution with AMIMBr were individually prepared. When the two solutions were mixed in the desired ratios at 100 °C, the homogeneous solutions were obtained, which could be converted into the binary ion gels by standing at room temperature for four days, followed by washing with ethanol. The resulting gels were characterized by the XRD and TGA measurements, which suggested relative good compatibility among cellulose, chitin, and ILs. The mechanical properties under compressive mode were changed depending on the cellulose/chitin ratios. The binary ion gels were further converted into cellulose/chitin binary films as followed [65,66]. The cellulose/chitin homogeneous solutions with ILs were first casted on a glass plate and left standing at room temperature for 2 h. The resulting gel-like materials were then subjected to successive Soxhlet extractions with ethanol for 12 h and with water for 12 h, and subsequently dried to give the binary films.

The cellulose/chitin binary ion gel was employed as the novel electrolyte for an electric double layer capacitor (EDLC) [38,67,68]. First, the binary ion gel was treated with 2.0 mol/L H_2_SO_4_ aqueous solution for 3 h. Electrochemical characteristics of the resulting acidic cellulose/chitin binary gel electrolyte were investigated by galvanostatic charge-discharge measurements. The test cell with the binary gel electrolyte showed a specific capacitance of 162 F/g at room temperature, which was higher than that for a cell with an ordinal H_2_SO_4_ electrolyte (155 F/g). The binary gel electrolyte showed an excellent high-rate discharge capability in a wide range of current densities as well as H_2_SO_4_ aqueous solution. In addition, the discharge capacitance of the test cell retained over 80% of its initial value in 10^5^ cycles even at a high current density of 5000 mA/g. The above results indicated that the acidic cellulose/chitin binary gel electrolyte had a practical applicability to an advanced EDLC with the excellent stability and working performance.

Cellulose/carrageenan binary ion gels with BMIMCl could be obtained by the same procedure as that for the carrageenan ion gels [47]. For the fabrication of the binary ion gels, cellulose and carrageenan (15% w/w each) were first mixed and solubilized with BMIMCl by heating at 100 °C for 10 h with stirring. When the resulting homogeneous solutions were cooled to room temperature, they could suitably be handled after 12 h of standing, followed by treatment with acetone and ethanol, giving rise to binary ion gels. The mechanical properties of the binary ion gels were better than those of the cellulose ion gel with BMIMCl. The presence of the imidazolium-sulfate ion pairs on carrageenan chains, which were produced by the ion-exchange of sulfate metal cations in the original carrageenans with BMIMCl during the gelation, probably contributed to enhancement of the mechanical properties of the resulting ion gels.

When solutions of cellulose/xanthan gum with BMIMCl, which were prepared by heating the mixtures at 100 °C for 9 h, were left standing at room temperature for one day, they were totally tuned into binary ion gels without any further procedure, as same as that for the aforementioned xanthan gum ion gel [69]. The resulting binary ion gels, which were thinly prepared on a Petri dish, were subjected to Soxhlet extraction with ethanol for regeneration, followed by drying under ambient conditions to fabricate cellulose/xanthan gum binary films. The XRD profiles of the resulting films indicated relative compatibility of the two polysaccharides. With increasing the weight ratios of cellulose to xanthan gum, the mechanical properties of the films under tensile mode were hardened.

Cellulose/xanthan gum binary hydrogels were fabricated by soaking the binary ion gels in water according to the similar procedure for the production of the xanthan gum hydrogel as mentioned above. The water contents of the binary hydrogels were always higher than 90% regardless the cellulose/xanthan gum weight rations. Their compressive testing resulted in the similar tendency on the mechanical properties as that of the binary films, in which the higher weight ratios of cellulose to xanthan gum gave the harder binary hydrogels.

## 6. Conclusions

In this review, we presented that the polysaccharide ion gels were facilely fabricated from their solutions with ILs, *i.e.*, BMIMCl and AMIMBr. Furthermore, the ion gels have been converted into value-added sustainable materials, such as films and hydrogels, by appropriate procedures. The analytical results of the ion gels observed the interesting information. The viscoelastic studies on IL solutions in a pregel state provided information of polysaccharides, such as the molecular weight. Although the ion gels with multiple functions and unique properties have been fabricated from anionic polysaccharides, neutral polysaccharides have been also found to suitably give ion gels with ILs. Because natural polysaccharides are one of the most abundant organic resources and show a wide range of functions owing to their diverse structures, the materials obtained by several approaches described in this reviews have potential for practical applications in medical, tissue engineering, and environmentally benign fields in the future.

## References

[1] Schuerch C., Mark H.F., Bilkales N., Overberger C.G. (1986). Polysaccharides. Encyclopedia of Polymer Science and Engineering.

[2] Rouilly A., Rigal L. (2002). Agro-materials: A bibliographic review. J. Macromol. Sci. Part C Polym. Rev..

[3] Mohanty A.K., Misra M., Drzal L.T. (2002). Sustainable bio-composites from renewable resources: Opportunities and challenges in the green materials world. J. Polym. Environ..

[4] Lenz R.W. (1993). Biodegradable polymers. Adv. Polym. Sci..

[5] Klemm D., Heublein B., Fink H.-P., Bohn A. (2005). Cellulose: Fascinating biopolymer and sustainable raw material. Angew. Chem. Int. Ed..

[6] Kurita K. (2006). Chitin and chitosan: Functional biopolymers from marine crustaceans. Mar. Biotechnol..

[7] Rinaudo M. (2006). Chitin and chitosan: Properties and applications. Prog. Polym. Sci..

[8] Pillai C.K.S., Paul W., Sharma C.P. (2009). Chitin and chitosan polymers: Chemistry, solubility and fiber formation. Prog. Polym. Sci..

[9] Stephen A.M., Philips G.O., Williams P.A. (2006). Food Polysaccharides and Their Applications.

[10] El Seoud O.A., Koschella A., Fidale L.C., Dorn S., Heinze T. (2007). Applications of ionic liquids in carbohydrate chemistry: A windows of opportunities. Biomacromolecules.

[11] Feng L., Chen Z.I. (2008). Research progress on dissolution and functional modification of cellulose in ionic liquids. J. Mol. Liq..

[12] Liebert T., Heinze T. (2008). Interaction of ionic liquids with polysaccharides 5. Solvents and reaction media for the modification of cellulose. BioResources.

[13] Pinkert A., Marsh K.N., Pang S., Staiger M.P. (2009). Ionic liquids and their interaction with cellulose. Chem. Rev..

[14] Zakrzewska M.E., Bogel-Lukasik E., Bogel-Lukasik R. (2010). Solubility of carbohydrates in ionic liquids. Energy Fuels.

[15] Swatloski R.P., Spear S.K., Holbrey J.D., Rogers R.D. (2002). Dissolution of cellulose with ionic liquids. J. Am. Chem. Soc..

[16] Welton T. (1999). Room-temperature ionic liquids. Solvents for synthesis and catalysis. Chem. Rev..

[17] Wasserscheid P., Keim W. (2000). Ionic liquids—New “solutions” for transition metal catalysis. Angew. Chem. Int. Ed..

[18] Plechkova N.V., Seddon K.R. (2008). Applications of ionic liquids in the chemical industry. Chem. Soc. Rev..

[19] Graenacher C. (1934). Cellulose Solution. U.S. Patent.

[20] Maki-Arvela P., Anugwom I., Virtanen P., Sjoholm R., Mikkola J.P. (2010). Dissolution of lignocellulosic materials and its constituents using ionic liquids—A review. Ind. Crop. Prod..

[21] Gericke M., Fardim P., Heinze T. (2012). Ionic liquids—Promising but challenging solvents for homogeneous derivatization of cellulose. Molecules.

[22] Yang X., Wang Q., Yu H. (2014). Dissolution and regeneration of biopolymers in ionic liquids. Russ. Chem. Bull..

[23] Isik M., Sardon H., Mecerreyes D. (2014). Ionic liquids and cellulose: Dissolution, chemical modification and preparation of new cellulosic materials. Int. J. Mol. Sci..

[24] Kadokawa J., Kokorin A. (2011). Preparation of polysaccharide-based materials compatibilized with ionic liquids. Ionic Liquids: Application and Perspectives.

[25] Kadokawa J., Mun J., Sim H. (2012). Preparation of functional Ion gels of polysaccharides with ionic liquids. Handbook of Ionic Liquids: Properties, Applications and Hazards.

[26] Yamamoto K., Kadokawa J. (2013). Preparation of polysaccharide-based materials using ionic liquids. Kobunshi Ronbunshu.

[27] Kadokawa J., Murakami M., Kaneko Y. (2008). A facile preparation of gel materials from a solution of cellulose in ionic liquid. Carbohydr. Res..

[28] Kadokawa J. (2013). Ionic liquid as useful media for dissolution, derivatization, and nanomaterial processing of chitin. Green Sustain. Chem..

[29] Kadokawa J., Chan C.H., Chia C.H., Thomas S. (2014). Preparation of chitin-based nanofibrous and composite materials using ionic liquids. Physical Chemistry of Macromolecules: Macro to Nanoscales.

[30] Kadokawa J. (2015). Fabrication of nanostructured and microstructured chitin materials through gelation with suitable dispersion media. RSC Adv..

[31] Wang W.T., Zhu J., Wang X.L., Huang Y., Wang Y.Z. (2010). Dissolution behavior of chitin in ionic liquids. J. Macromol. Sci. Part B Phys..

[32] Muzzarelli R.A.A. (2011). Biomedical exploitation of chitin and chitosan via mechano-chemical disassembly, electro spinning, dissolution in imidazolium ionic liquids, and supercritical drying. Mar. Drugs.

[33] Jaworska M.M., Kozlecki T., Gorak A. (2012). Review of the application of ionic liquids as solvents for chitin. J. Polym. Eng..

[34] Bochek A.M., Murav’ev A.A., Novoselov N.P., Zaborski M., Zabivalova N.M., Petrova V.A., Vlasova E.N., Volchek B.Z., Lavrent’ev V.K. (2012). Specific features of cellulose and chitin dissolution in ionic liquids of varied structure and the structural organization of regenerated polysaccharides. Russ. J. Appl. Chem..

[35] Wu Y., Sasaki T., Irie S., Sakurai K. (2008). A novel biomass-ionic liquid platform for the utilization of native chitin. Polymer.

[36] Qin Y., Lu X., Sun N., Rogers R.D. (2010). Dissolution or extraction of crustacean shells using ionic liquids to obtain high molecular weight purified chitin and direct production of chitin films and fibers. Green Chem..

[37] Vygodskii Y.S., Lozinskaya E.L., Shaplov A.S. (2002). Ionic liquids as novel reaction media for the synthesis of condensation polymers. Macromol. Rapid Commun..

[38] Yamazaki S., Takegawa A., Kaneko Y., Kadokawa J., Yamagata M., Ishikawa M. (2009). An acidic cellulose-chitin hybrid gel as novel electrolyte for an electric double layer capacitor. Electrochem. Commun..

[39] Prasad K., Murakami M., Kaneko Y., Takada A., Nakamura Y., Kadokawa J. (2009). Weak gel of chitin with ionic liquid, 1-allyl-3-methylimidazolium bromide. Int. J. Biol. Macromol..

[40] Kadokawa J., Takegawa A., Mine S., Prasad K. (2011). Preparation of chitin nanowhiskers using an ionic liquid and their composite materials with poly(vinyl alcohol). Carbohydr. Polym..

[41] Tajiri R., Setoguchi T., Wakizono S., Yamamoto K., Kadokawa J. (2013). Preparation of self-assembled chitin nanofibers by regeneration from ion gels using calcium halide•dihydrate/methanol solutions. J. Biobased Mater. Bioenergy.

[42] Hatanaka D., Yamamoto K., Kadokawa J. (2014). Preparation of chitin nanofiber-reinforced carboxymethyl cellulose films. Int. J. Biol. Macromol..

[43] Setoguchi T., Yamamoto K., Kadokawa J. (2012). Preparation of chitin nanofiber-graft-poly(l-lactide-*co*-ε-caprolactone) films by surface-initiated ring-opening graft copolymerization. Polymer.

[44] Kadokawa J., Setoguchi T., Yamamoto K. (2013). Preparation of highly flexible chitin nanofiber-*graft*-poly(γ-l-glutamic acid) network film. Polym. Bull..

[45] Yamamoto K., Yoshida S., Kadokawa J. (2014). Surface-initiated atom transfer radical polymerization from chitin nanofiber macroinitiator film. Carbohydr. Polym..

[46] Van de Velde F., de Ruiter G.A., Steinbüchel A., Rhee S.K. (2005). Carrageenan, In Polysaccharides and Polyamides in the Food Industry.

[47] Prasad K., Kaneko Y., Kadokawa J. (2009). Novel gelling systems of κ-, λ- and ι-carrageenans and their composite gels with cellulose using ionic liquid. Macromol. Biosci..

[48] Jansson P.E., Kenne L., Lindberg B. (1975). Structure of the extracellular polysaccharide from *Xanthomonas*
*campestris*. Carbohydr. Res..

[49] Izawa H., Kaneko Y., Kadokawa J. (2009). Unique gel of xanthan gum with ionic liquid and its conversion into high performance hydrogel. J. Mater. Chem..

[50] Izawa H., Kadokawa J. (2010). Preparation and characterizations of functional ionic liquid-gel and hydrogel of xanthan gum. J. Mater. Chem..

[51] Dea I.C.M., Morrison A. (1975). Chemistry and interactions of seed galactomannans. Adv. Carbohydr. Chem. Biochem..

[52] Prasad K., Izawa H., Kaneko Y., Kadokawa J. (2009). Preparation of temperature-induced shapeable film material from guar gum-based gel with an ionic liquid. J. Mater. Chem..

[53] Mine S., Prasad K., Izawa H., Sonoda K., Kadokawa J. (2010). Preparation of guar gum-based functional materials using ionic liquid. J. Mater. Chem..

[54] Kadokawa J., Kato T., Setoyama M., Yamamoto K. (2013). Preparation of galactomannan-based materials compatibilized with ionic liquids. J. Polym. Environ..

[55] Izawa H., Wakizono S., Kadokawa J. (2010). Fluorescence resonance-energy-transfer in systems of Rhodamine 6G with ionic liquid showing emissions by excitation at wide wavelength areas. Chem. Commun..

[56] Takada A., Takahashi Y. (2007). Solution properties of cellulose in ionic liquids. Cellulose Commun..

[57] Kawahara K., Ohta K., Miyamoto H., Nakamura S. (1984). Preparation and solution properties of pullulan fractions as standard samples for water-soluble polymers. Carbohydr. Polym..

[58] Caltey B.J., Whelan W.J. (1971). Observations on the structure of pullulan. Arch. Biochem. Biophys..

[59] Hu H., Kamo M., Takada A., Takahashi Y. (2010). Viscoelasticity of pullulan in ionic liquid BmimCl. Proceedings of the of 5th Pacific Rim Conference on Rheology.

[60] Ferry J.D. (1980). Viscoelastic Properties of Polymers.

[61] Osaki K., Inoue T., Uematsu T. (2001). Viscoelastic properties of dilute polymer solutions: The effect of varying the concentration. J. Polym. Sci..

[62] Osaki K., Inoue T., Uematsu T., Yamashita Y. (2002). Rheology of polystyrene solutions around the coil overlapping concentration: A phenomenological description of stresses in simple shear flow. J. Polym. Sci..

[63] Takada A., Nakamura M., Takahashi Y. Viscoelastic properties of cellulose solution in an ionic liquid 1-butyl-3-methylimidazolium chloride. Proceedings of the 2010 International Symposium on Non-Equilibrium Soft Matter.

[64] Kadokawa J., Murakami M., Takegawa A., Kaneko Y. (2009). Preparation of cellulose-starch composite gel and fibrous material from a mixture of the polysaccharides in ionic liquid. Carbohydr. Polym..

[65] Takegawa A., Murakami M., Kaneko Y., Kadokawa J. (2010). Preparation of chitin/cellulose composite gels and films with ionic liquids. Carbohydr. Polym..

[66] Kadokawa J., Hirohama K., Mine S., Kato T., Yamamoto K. (2012). Facile preparation of chitin/cellulose composite films using ionic liquids. J. Polym. Environ..

[67] Yamazaki S., Takegawa A., Kaneko Y., Kadokawa J., Yamagata M., Ishikawa M. (2010). Performance of electric double-layer capacitor with acidic cellulose-chitin hybrid gel electrolyte. J. Electrochem. Soc..

[68] Yamazaki S., Takegawa A., Kaneko Y., Kadokawa J., Yamagata M., Ishikawa M. (2010). High/low temperature operation of electric double layer capacitor utilizing acidic cellulose-chitin hybrid gel electrolyte. J. Power Sourc..

[69] Setoyama M., Yamamoto K., Kadokawa J. (2014). Preparation of cellulose/xanthan gum composite films and hydrogels using ionic liquid. J. Polym. Environ..

